# Neuroprotection After Intracerebral Hemorrhage: Apoptosis, Neurogenesis, and Prospects of Stem Cell Therapy

**DOI:** 10.1155/sci/4696038

**Published:** 2026-06-19

**Authors:** Ziwei Wang, Jing Tian, Ying Kong

**Affiliations:** ^1^ Second Clinical Medical College, Heilongjiang University of Chinese Medicine, Harbin, 150000, Heilongjiang Province, China, hljucm.edu.cn; ^2^ Rehabilitation Medicine and Traditional Chinese Medicine Department, Huai’an Huai’an Hospital, Huai’an, 223200, Jiangsu Province, China; ^3^ Acupuncture Department, The Second Affiliated Hospital of Heilongjiang University of Chinese Medicine, Harbin, 150000, Heilongjiang Province, China, hljucm.edu.cn

**Keywords:** apoptosis, future perspectives, intracerebral hemorrhage, neurogenesis, neuroprotection, stem cell therapy

## Abstract

This comprehensive review examines the interconnected roles of apoptosis, neurogenesis, and stem cell therapy in neuroprotection and neurorestoration following intracerebral hemorrhage (ICH). We emphasize that these processes should be interpreted within a unified secondary injury cascade rather than as independent topics. Following ICH, hematoma toxicity, erythrocyte lysis, iron overload, oxidative stress, inflammation, blood‐brain barrier disruption, and perihematomal edema interact to activate multiple regulated cell death pathways. Apoptosis remains important, but ferroptosis and other inflammatory cell–death programs are also increasingly relevant to ICH research. The endogenous neurogenic response following ICH is analyzed, highlighting both its potential and its marked limitations in spontaneous recovery, particularly the poor survival and functional integration of newborn cells. We then examine how stem cell therapy may bridge antiapoptotic neuroprotection and proneurogenic neurorestoration through paracrine signaling, microenvironment modulation, and limited cell replacement. Current diagnostic advances and clinical trial designs for stem cell therapy in ICH are reviewed, but we also discuss major translational barriers, including weak distinction between rodent and human evidence, limited cell survival, delivery‐route constraints, immune compatibility, source heterogeneity, and tumorigenicity. Finally, we propose that future progress will require stage‐specific and clinically translatable strategies rather than isolated pathway targeting. This integrated framework may help refine therapeutic priorities for ICH patients.

## 1. Introduction

Intracerebral hemorrhage (ICH) is a severe cerebrovascular disorder characterized by high mortality and disability. The pathophysiological mechanisms underlying ICH are complex, and the initial hematoma is followed by a secondary injury cascade involving thrombin toxicity, erythrocyte lysis, iron overload, oxidative stress, inflammation, blood‐brain barrier disruption, and progressive cell death [1]. Within this cascade, apoptosis plays a critical role, and molecules such as alpha2delta‐1 and BID may participate in post‐ICH injury signaling [2, 3]. However, these pathways should be interpreted in the context of interacting neurovascular processes rather than as isolated molecular events.

The selection of apoptosis, neurogenesis, and stem cell therapy as the focus of this review is based on their interconnected roles in ICH pathophysiology and repair. Following ICH, the initial hematoma triggers a cascade of secondary injury mechanisms, among which apoptosis represents a predominant mode of delayed neuronal death that continues for days to weeks after the initial insult. Understanding and modulating apoptotic pathways are therefore critical for limiting the expansion of brain injury. Concurrently, the injured brain exhibits an endogenous regenerative response characterized by enhanced neurogenesis in specific neurogenic niches, though this spontaneous repair capacity is often insufficient for functional recovery. Stem cell therapy emerges as a promising strategy that bridges these two processes: transplanted stem cells not only secrete antiapoptotic factors that reduce ongoing cell death but also enhance endogenous neurogenesis through paracrine mechanisms and may partially support tissue repair. Thus, these three areas form a coherent framework—from limiting damage (antiapoptosis) to enhancing repair (neurogenesis promotion) to therapeutic intervention (stem cell transplantation)—that encompasses the continuum of neuroprotection and neurorestoration in ICH. Nevertheless, the strength of evidence differs across these domains, and findings from rodent models should not be directly equated with human recovery.

Moreover, ferroptosis, a newly recognized form of cell death, has attracted significant attention regarding its role in ICH. Studies have shown that post‐ICH abnormalities in iron metabolism, lipid peroxidation, and related neuroinflammation may promote ferroptotic injury, and inhibition of ferroptosis could represent a novel therapeutic approach for treating ICH [4]. Furthermore, interactions between autophagy and apoptosis have been identified, with Beclin 1, Bax, and Bcl‐2 playing pivotal roles [5]. Emerging evidence also suggests that pyroptotic signaling may contribute to the inflammatory microenvironment after ICH because induced neural stem cells can suppress the microglial pyroptotic pathway in experimental models [6]. Necroptosis is also increasingly discussed in ICH research, underscoring that apoptosis is only one component of a broader regulated cell–death network.

Historically, it was believed that the mammalian central nervous system lacked the ability to repair itself; however, an increasing body of evidence suggests that there is a certain degree of reparative capacity following injury [7]. In ICH, endogenous neurogenic responses have been described in the subventricular zone and dentate gyrus, but the spontaneous response appears insufficient for complete functional recovery. Additionally, histone 3 lysine 27 (H3K27) demethylases, Kdm6a and Kdm6b, may influence mammalian neural regeneration, suggesting that epigenetic regulation could modify the neurogenic niche after brain injury [8]. At present, however, most of this evidence remains preclinical.

Nerve growth factor (NGF) has neuroprotective and regenerative effects in cerebral ischemic recovery. It inhibits neuronal apoptosis, reduces inflammatory responses, promotes axonal regeneration, and enhances angiogenesis by interacting with the TrkA receptor [9]. Although these mechanisms are not specific to ICH, they illustrate how trophic support may shape the posthemorrhagic reparative milieu. Meanwhile, stem cells derived from human clinical waste have been proposed as a potentially accessible source for regenerative medicine [10], but their value in ICH should still be regarded as investigational rather than established.

Stem cell therapy offers new hope for the treatment of ICH because stem cells can secrete multiple cytokines and may modify apoptosis, inflammation, angiogenesis, and endogenous repair [11, 12]. However, stem cell therapy should not be described in overly optimistic terms. In hemorrhagic stroke, the dominant evidence supports paracrine microenvironmental modulation more strongly than direct neuronal replacement, and [12] most supporting data still come from preclinical models.

In addition, biomaterial engineering and theoretical frameworks may eventually improve stem cell recruitment, survival, and functional integration [13–15]. Nevertheless, concepts derived from other tissues or diseases cannot be translated directly to ICH. Therefore, this review focuses on how apoptosis, endogenous neurogenesis, and stem cell–based interventions intersect within the specific pathophysiology of ICH.

## 2. Epidemiology and Current Status of ICH

### 2.1. Epidemiological Features of ICH

ICH is a significant global health issue, with its epidemiological characteristics influenced by various factors. Studies indicate that the global incidence rate of ICH is 29.9 per 100,000 person‐years (95% CI: 26.5–33.3), with a notably higher incidence observed in Asian populations compared to other continents [16]. The incidence increases with age, with a notable threshold at 85 years. Furthermore, males are more likely to suffer from ICH than females, and the basal ganglia are the most common site of hemorrhage.

In terms of risk factors, hypertension is the most significant contributor to ICH, while excessive alcohol consumption, cardiovascular diseases, and other conditions are also strongly associated with its occurrence. For patients undergoing dialysis, the incidence of ICH is higher, with worse clinical outcomes. The use of dialysis and anticoagulant medications is an important risk factor for hematoma expansion [17]. Additionally, factors such as blood glucose levels, hematoma location, volume, and whether craniotomy is performed are associated with the development of posthemorrhagic seizures. The occurrence of seizures is, in turn, linked to poor functional outcomes 2 years after the hemorrhage [18].

### 2.2. The Role of Apoptosis in ICH

**Figure 1:**
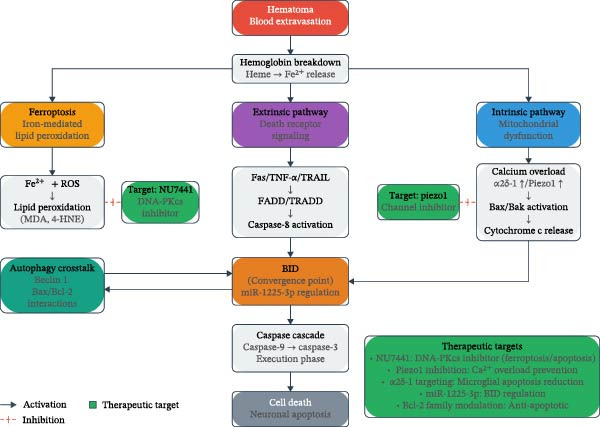
Representative apoptosis‐centered and ferroptotic pathways activated after ICH. The initial hematoma triggers multiple cell death pathways including iron‐mediated ferroptosis (left, orange), death receptor‐mediated extrinsic pathway (center, purple), and mitochondrial intrinsic pathway (right, blue). Key regulatory proteins (alpha2delta‐1, Piezo1, and BID) represent potential therapeutic targets shown in green boxes. The ferroptosis pathway is initiated by iron release from hemoglobin breakdown, leading to lipid peroxidation and cell death. The extrinsic pathway involves death receptor activation leading to caspase‐8 activation. The intrinsic pathway is triggered by calcium overload through upregulated alpha2delta‐1 and Piezo1 channels, causing mitochondrial dysfunction and cytochrome c release. BID serves as a convergence point integrating signals from multiple pathways and is regulated by miR‐1225‐3p. The crosstalk between autophagy and apoptosis through Beclin 1, Bax, and Bcl‐2 interactions further modulates cell fate decisions. These pathways should be interpreted as part of the broader inflammatory, oxidative, and neurovascular injury cascade after ICH.

Apoptosis plays a critical role in secondary brain injury following ICH, as summarized in Figure 1. However, apoptosis should be interpreted within a broader secondary injury cascade that also includes inflammation, oxidative stress, blood‐brain barrier disruption, and perihematomal edema. Studies have shown that several apoptosis‐related mechanisms are activated after ICH. For example, calcium overload mediated by alpha2delta‐1 can induce endoplasmic reticulum stress and apoptosis in BV2 microglial cells. Silencing alpha2delta‐1 inhibits this process, suggesting that alpha2delta‐1 may be a potential therapeutic target for ICH. Furthermore, gene analysis in ICH patients has identified BID as a key gene in apoptosis, playing a significant role in post‐ICH apoptotic regulation, with miR‐1225‐3p potentially involved in the regulation of BID expression.

Additionally, ferroptosis, which is closely linked to iron overload after hemorrhage, has been found to be associated with ICH. Post‐ICH iron metabolism abnormalities and lipid peroxidation can trigger ferroptosis, leading to neuronal death. Inhibition of ferroptosis may therefore alleviate brain injury following ICH. Some drugs, such as NU7441, can reduce neuronal apoptosis, oxidative stress, and ferroptosis by inhibiting DNA‐PKcs, thus mitigating ICH‐induced damage [19]. Moreover, Piezo1, a mechanosensitive calcium channel, is highly expressed in oligodendrocytes after ICH. Inhibition of Piezo1 can reduce brain damage, decrease oligodendrocyte apoptosis, and improve neurological function [20]. Experimental work also suggests that pyroptotic signaling contributes to secondary injury after ICH, further supporting the view that multiple regulated cell–death mechanisms coexist in the hemorrhagic brain [6].

**Figure 2:**
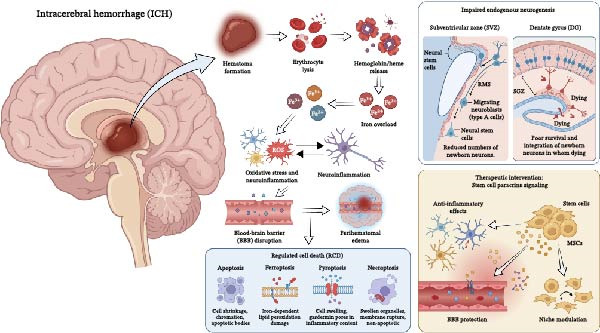
Integrated secondary injury and neurorepair framework after ICH. Hematoma formation initiates thrombin signaling, erythrocyte lysis, iron overload, oxidative stress, inflammation, blood‐brain barrier disruption, and perihematomal edema. These processes interact to activate apoptosis, ferroptosis, pyroptosis, and necroptosis, leading to neurovascular injury and constraining endogenous neurogenesis. Stem cell–based strategies are hypothesized to intervene mainly by modulating the microenvironment rather than by direct large‐scale cell replacement.

Beyond these specific targets, multiple apoptotic pathways are activated following ICH. The extrinsic pathway, mediated by death receptors such as Fas and TNF receptors, and the intrinsic (mitochondrial) pathway, regulated by Bcl‐2 family proteins, both contribute to neuronal death after ICH. BID, identified as a key gene in post‐ICH apoptosis through genetic analysis, serves as a bridge between these pathways, with miR‐1225‐3p potentially regulating its expression [3]. The temporal pattern of apoptosis activation shows an early peak within 24–48 h, followed by sustained activation for up to 2 weeks post‐ICH, providing multiple therapeutic windows for intervention. Furthermore, the crosstalk between autophagy and apoptosis, particularly through Beclin 1, Bax, and Bcl‐2 interactions, adds another layer of complexity to cell fate determination after ICH [5]. These pathways do not act in isolation; instead, they interact with inflammation, oxidative stress, and blood‐brain barrier damage within the broader secondary injury cascade (Figure 2).

### 2.3. Current Status of Neuroregeneration After ICH

**Figure 3:**
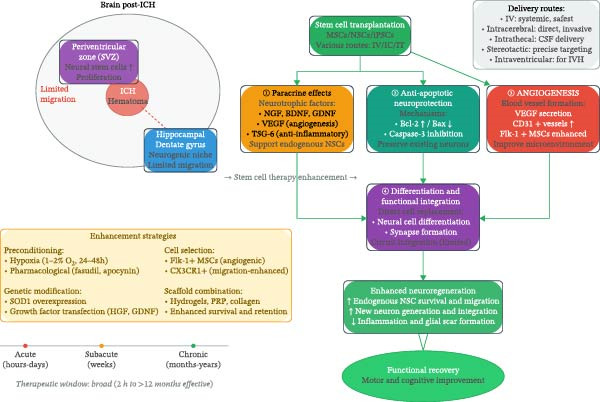
Endogenous neurogenesis occurs in specific brain regions following ICH but remains insufficient for complete repair. The left panel shows the brain post‐ICH with hematoma (red) and two main neurogenic niches: the periventricular zone/subventricular zone (SVZ, purple) and hippocampal dentate gyrus (blue). Following ICH injury, endogenous neural stem cells in these regions exhibit increased proliferation, but their migration to injury sites is limited (red dashed arrows), resulting in inadequate spontaneous repair.

Multiple interventional strategies have been investigated to enhance neuroregeneration after ICH, as summarized in Figure 3. Early physical activity promotes sensory–motor function recovery in experimental ICH models by preventing neuronal death, cortical atrophy, and dendritic loss while inhibiting neuroinflammation [21]. However, most available evidence for enhanced neuroregeneration after ICH remains preclinical.

Endogenous neurogenic responses following ICH have been documented in periventricular regions and the hippocampal dentate gyrus, where neural stem/progenitor cells exhibit increased proliferation in response to the injury. However, the survival and functional integration of these newly generated neurons remain limited. Acupuncture at specific acupoints (Baihui DU20 and Qubin GB7) effectively reduces hemorrhage and brain edema while upregulating neurotrophic factors including Nestin and bFGF, thereby promoting functional recovery [22]. Emerging evidence suggests that modulating the immune microenvironment can enhance neuroregeneration, and shifting microglia, macrophages, and T lymphocyte subtypes toward anti‐inflammatory and regenerative phenotypes may improve ICH prognosis [23]. Additionally, the upregulation of H3K27 demethylases (Kdm6a and Kdm6b) has been implicated in regulating neural regeneration capacity, suggesting epigenetic modifications as potential therapeutic targets. Nevertheless, current evidence for clinically meaningful neurogenesis in adult human ICH remains limited, and rodent findings should not be overinterpreted as proof of functional human neuroregeneration.

## 3. Advances in Diagnostic Techniques After ICH

### 3.1. Detection Technologies for Apoptosis Biomarkers

Accurate detection of apoptosis biomarkers is crucial for understanding the pathological processes of ICH and evaluating treatment efficacy. Traditional detection methods, such as those based on flow cytometry and fluorescence microscopy, are widely used; however, they have limitations including high equipment costs and complex operation, making them suitable only for specialized laboratories with trained personnel [24]. Novel microfluidic devices have emerged that can detect the externalization of phosphatidylserine, a key biomarker of apoptosis on the cell membrane. These devices convert single capture events into electrical signals, enabling electronic detection of apoptotic cells. This technology has been validated in simulated samples and holds potential for application in various settings, providing a new approach for apoptosis detection [24].

Additionally, apoptosis can be effectively differentiated into live cells, early apoptotic cells, late apoptotic cells, and necrotic cells through chemical treatments and acidic pH–induced apoptosis, followed by Annexin V flow cytometry [25]. Different cell harvesting methods can significantly impact the detection of cell surface proteins and apoptosis biomarkers, requiring careful selection of appropriate cell separation techniques based on the markers being analyzed to avoid experimental bias [26]. Furthermore, methods based on fluorescence detection of caspase activation allow for real‐time, high‐throughput, and low‐cost apoptosis detection in vitro, providing more convenient tools for apoptosis research [27].

### 3.2. Imaging Advancements in Neuroregeneration Assessment

The continuous development of imaging technologies has provided strong support for assessing neuroregeneration. Various imaging modalities such as computed tomography (CT), magnetic resonance imaging (MRI), positron emission tomography (PET), and angiography are widely applied in the diagnosis and assessment of cerebrovascular diseases, each with its strengths and limitations [28]. For example, CT offers rapid imaging and is used for the initial diagnosis of acute ICH but has a relatively low resolution for soft tissues. MRI, on the other hand, provides high‐resolution imaging of neural soft tissues and offers more detailed structural information.

Emerging imaging techniques such as perfusion imaging, diffusion tensor imaging (DTI), and molecular imaging have introduced new opportunities for neuroregeneration assessment. Perfusion imaging assesses the blood flow in brain tissue, which helps to understand changes in blood supply during neuroregeneration. DTI shows the orientation and integrity of neural fibers, providing important information on structural changes during neuroregeneration. Molecular imaging can visualize biological processes related to neuroregeneration by labeling specific molecules, such as tracking neural stem cells and detecting neurotransmitters [28]. The application of these novel technologies helps to more accurately assess the progression and effectiveness of neuroregeneration, providing more precise guidance for clinical treatment.

### 3.3. Clinical Trial Design for Stem Cell Therapy

The design of clinical trials for stem cell therapy is critical for assessing safety and efficacy. As of December 2024, there are 116 human pluripotent stem cell (hPSC) interventional trials approved by regulatory agencies globally, testing 83 types of hPSC products. The majority of these trials target diseases related to the eyes, central nervous system, and cancer, with more than 1200 patients receiving hPSC product treatments and over 10 clinical doses administered, as summarized in Table 1 [45]. However, these broad hPSC data should not be interpreted as established efficacy in ICH, where human studies remain in the early phase and relatively small.

**Table 1:** Summary of representative preclinical and clinical stem cell studies for intracerebral hemorrhage and their main limitations.

A. Representative preclinical studies							
Study	Species/model	Cell type	Delivery route	Timing	Cell dose	Key outcomes	Limitations
Chen et al. [29]	Rat, collagenase‐induced ICH	Bone marrow MSCs	Intravenous	2 h post‐ICH	Not specified	Reduced BBB disruption, neurological deficits, brain edema; TSG‐6–mediated NF‐κB suppression	72 h follow‐up only, optimal dosing undefined
Choi et al. [30]	Rat, collagenase‐induced striatal ICH	Human placenta–derived MSCs	Intravenous	1 h post‐ICH	1 × 10^6^ cells	Mortality reduction (37.5%→12.5%), decreased hematoma volume, reduced neuronal death	24 h observation only, mechanism unclear
Duan et al. [31]	Rat, collagenase‐induced ICH	MSC‐derived exosomes (miR‐146a‐5p)	Intravenous	24 h post‐ICH	100 μg/mL	Reduced apoptosis, inhibited M1 polarization, targeted IRAK1/NFAT5	28‐day follow‐up, manufacturing scalability unclear
Liu et al. [32]	Mouse, collagenase‐induced ICH	Hypoxic‐preconditioned OM‐MSCs	Intracerebral	24 h post‐ICH	5 × 10^5^ cells	Superior recovery vs. normoxic MSCs, enhanced survival, miR‐326–mediated autophagy	Species mismatch (rat→mouse), 28‐day follow‐up
Bao et al. [33]	Rat, collagenase‐induced ICH	Flk‐1+ human BM‐MSCs	Intracerebral	Early post‐ICH	2 × 10^5^ cells	Enhanced angiogenesis, anti‐inflammation, improved behavior	Xenogeneic transplant, engraftment undefined
Lee et al. [34]	Rat, collagenase‐induced ICH	Human fetal NSCs (H1 clone)	IV or intracerebral	2 h or 24 h post‐ICH	5M (IV) or 1M (IC)	Only IV‐2 h effective; spleen‐mediated immunomodulation	Narrow 2 h window, minimal brain engraftment
Wakai et al. [35]	Mouse, autologous blood ICH	SOD1‐overexpressing NSCs	Intracerebral	3 days post‐ICH	Not specified	Enhanced NSC survival, reduced atrophy, improved behavior at 35 days	Mouse model only, genetic modification required
Liu et al. [6]	Rat, collagenase‐induced ICH	Induced NSCs from placental MSCs	Intracerebral	Not specified	Not specified	Suppressed pyroptosis, M1→M2 shift, no teratomas	Limited details in abstract, translation pathway unclear
Qin et al. [36]	Rat, collagenase‐induced striatal ICH	Rat skin–derived iPSCs	Intracerebral (via needle tract)	6 h post‐ICH	1 × 10^6^ cells	Reduced inflammation (MPO↓ and CD11b↓), apoptosis↓, 83% survival vs. 67%	Tumorigenicity concern (though none seen at 42 days)
Suda et al. [37]	Rat (young and aged), autologous blood ICH	Autologous BM‐MNCs	Intravenous	24 h post‐ICH	1 × 10^7^ cells/kg	Broad short/long‐term benefits in both young and aged	Heterogeneous cell population, mechanisms unclear

B. Completed clinical trials							
Study	Phase	Cell type	*n*	Delivery route	Timing	Dose	Key safety outcomes
Durand et al. [38] (Mayo Clinic)	I	Allogeneic BM‐MSCs (GMP‐grade)	9	Intravenous	Acute phase	0.5, 1.0, and 2.0 M cells/kg (dose escalation)	0% mortality, no SAEs, no DLT; safe across all doses
Ahn et al.[39]	I	Allogeneic UCB‐MSCs	9 preterm infants	Intraventricular	Post‐IVH with hydrocephalus	1 × 10^7^ cells/kg (escalating)	Well‐tolerated, 0% mortality, no DLT
Li et al. [40]	I/II	Autologous BM‐MNCs	100 (60 Tx, 40 controls)	Intracerebral (via drainage tube)	Mean 5.9 days postevacuation	Variable	No new hemorrhage, no major complications, 5%–6% fever
Tsang et al. [41]	I/II	Autologous BM‐MSCs	9 chronic stroke (5 Tx, 4 placebo; ICH subset)	Intravenous (2 injections and 4 weeks apart)	1 year poststroke (chronic)	From 29 mL BM (mean)	Safe, no SAEs, well‐tolerated
Zhu et al. [42]	Retrospective cohort	Autologous BM‐MSCs	206 surgical ICH (110 Tx and 96 controls)	Combined: intracerebral + intrathecal	5 days (IC) and 4 weeks (IT) postevacuation	Mean 8.5 × 10^7^ cells	No transplant‐related AEs
Chang et al. [43]	Retrospective	Autologous BM‐MNCs + allogeneic UCB cells	60 (groups vs. controls)	Intracerebral (via drainage tube)	2–3 weeks postevacuation	Variable	No late AEs over 5 years

C. Ongoing/recruiting clinical trial							
Trial	Phase	Cell type	Target *n*	Delivery	Timing	Dose	Primary endpoint
RAINBOW‐Hx (Hokkaido) [44]	I/II	Autologous BMSCs + RCP scaffold (HUFF‐01)	8	Stereotactic intracerebral (MRI‐guided)	≥12 months post‐ICH (chronic)	50 M cells (5 × 10^7^) in scaffold	Safety (AEs at 12 months with DSMB)

Stem cell therapy has garnered significant attention in stroke rehabilitation. In ICH, several clinical studies are exploring cell type, dose, administration timing and route, patient selection criteria, and clinical outcomes [46]. Autologous bone marrow–derived mononuclear cells and mesenchymal stem cells are among the most commonly studied products, delivered intravenously in the acute‐to‐subacute phase or by intracerebral or intrathecal approaches in selected chronic settings [38, 40, 44]. Although early studies suggest feasibility and acceptable safety, definitive clinical efficacy remains to be established, and larger randomized, double‐blind, and placebo‐controlled trials are still required. Importantly, rodent efficacy data should not be overinterpreted as human proof of benefit.

## 4. Therapeutic Strategies After ICH

### 4.1. Pharmacological Research on Inhibiting Apoptosis

In the context of ICH, several pharmacological agents have shown promise in inhibiting apoptosis. NU7441, a selective DNA‐PKcs inhibitor, reduces neuronal apoptosis, oxidative stress, and ferroptosis following ICH, thereby mitigating brain injury. Inhibition of Piezo1, a mechanosensitive calcium channel highly expressed in oligodendrocytes after ICH, decreases oligodendrocyte apoptosis and improves neurological function. Additionally, targeting the alpha2delta‐1 protein, which is significantly upregulated in hemorrhagic brain tissue, can reduce calcium overload‐induced apoptosis in microglial cells. These ICH‐specific antiapoptotic strategies demonstrate the potential for targeted neuroprotection following hemorrhagic stroke, but single‐pathway inhibition alone may be insufficient because apoptosis interacts with ferroptosis, inflammation, and blood‐brain barrier injury.

### 4.2. Treatment Methods to Promote Neuroregeneration

Various treatment strategies aimed at promoting neuroregeneration offer hope for the rehabilitation of ICH patients. Potential approaches include early rehabilitation, modulation of the immune microenvironment, neurotrophic support, and stem cell–based paracrine therapies. Early exercise can reduce inflammation and support sensorimotor recovery in experimental ICH [21]. Acupuncture and other niche‐modulating interventions have been reported to upregulate neurotrophic factors and improve functional recovery in preclinical settings [22]. Immune reprogramming toward a prorepair peri‐injury milieu may further support neurogenesis and angiogenesis after hemorrhage [23, 47].

However, most neuroregenerative interventions remain preclinical, and efficacy is difficult to compare across studies because of heterogeneity in model type, timing, outcome measures, and follow‐up. Approaches developed for peripheral nerve injury or other tissues cannot be translated directly to ICH, where hematoma toxicity, blood‐brain barrier disruption, and perihematomal edema create a distinct microenvironment [48]. Therefore, treatment strategies that appear promising experimentally still require critical evaluation of reproducibility and translational relevance [49].

### 4.3. Clinical Applications of Stem Cell Therapy

**Figure 4:**
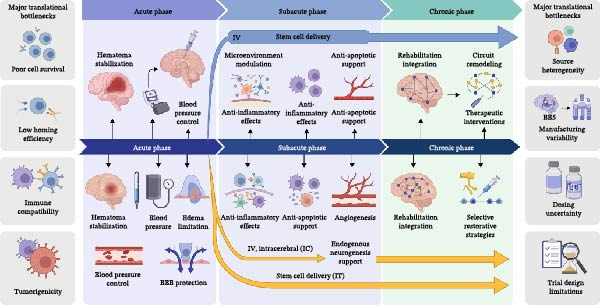
Translational roadmap for stem cell–based therapy after ICH. Effective repair requires phase‐specific management: acute stabilization of the hematoma and neurovascular unit, subacute microenvironment modulation, and later integration with rehabilitation and longer‐term restorative strategies. Key translational bottlenecks include route selection, cell survival, product heterogeneity, immune compatibility, tumorigenicity, manufacturing, and trial design.

Stem cell therapy has shown potential in the treatment of ICH and other neurological disorders, but the current evidence is weighted toward safety and feasibility rather than definitive efficacy. In ICH, major challenges include effective delivery, survival, and homing of transplanted cells; variability between stem cell sources; immune compatibility; tumorigenicity; and regulatory oversight [38–44, 50]. Ongoing work aims to optimize cell type, dose, timing, and route of administration, but clinical translation remains incomplete. The phase‐specific therapeutic rationale and major translational bottlenecks of stem cell therapy in ICH are summarized in Figure 4.

## 5. Controversies and Challenges After ICH

### 5.1. Safety Concerns in Apoptosis Inhibition

The safety of apoptosis‐modulating drugs is a significant concern in ICH because excessive inhibition of cell death pathways may interfere with the clearance of injured cells and may also affect physiological signaling in normal tissues. Experience from other disease fields shows that Bcl‐2‐ or IAP‐related interventions can produce meaningful biological effects, but these safety profiles cannot be directly extrapolated to hemorrhagic stroke [51, 52]. Therefore, antiapoptotic strategies in ICH should aim for stage‐specific modulation rather than indiscriminate suppression.

Moreover, apoptosis‐related proteins participate in multiple physiological processes, including cell survival, proliferation, and differentiation. Interfering with these pathways may trigger unintended adverse effects, especially if treatment is prolonged or delivered outside the optimal therapeutic window [52]. For ICH, the key issue is not simply whether apoptosis can be inhibited but how this intervention interacts with ferroptosis, inflammation, blood‐brain barrier disruption, and tissue repair.

### 5.2. Controversies Regarding the Effectiveness of Neuroregenerative Therapy

The effectiveness of neuroregenerative therapies remains controversial within the academic community. Experience from other neuroregenerative fields shows that experimental success does not guarantee clinical benefit [53–55]. In ICH, many interventions improve histology or short‐term behavioral outcomes [53] in rodents, but reproducibility, standardized endpoints, and durable functional gains remain uncertain [54].

However, the translation from animal experiments to clinical applications faces several challenges. Some neuroregenerative treatments that are effective in rodent models may fail in practice because of species differences, age and comorbidity mismatch, variability in hematoma location and volume, and the complexity of clinical rehabilitation. Furthermore, the lack of standardized criteria for evaluating efficacy across studies complicates the assessment of clinical relevance. Therefore, further research should focus not only on mechanism but also on model quality, study design, and clinically meaningful outcome measures [55].

### 5.3. Ethical and Regulatory Challenges in Stem Cell Therapy

While stem cell therapy shows potential in ICH, it also faces significant ethical and regulatory challenges. Ethically, the source of stem cells raises concerns, particularly with the acquisition of embryonic stem cells, which may provoke debates over the moral status of embryos [56]. Although induced pluripotent stem cells (iPSCs) avoid some ethical concerns associated with embryonic stem cells, they still pose potential risks, including tumor formation and other unintended consequences [57].

Regulatory challenges also arise because stem cell therapy is a complex biological product. Regulatory frameworks differ across countries and regions, and the lack of standardized guidelines presents difficulties in conducting international research and clinical applications of stem cell therapies [58]. Moreover, quality control of cell products is a crucial regulatory focus. Strict standards are required for cell sourcing, preparation, storage, and transportation to ensure safety and reproducibility, and regulatory oversight of clinical trial design and implementation must be strengthened to protect patients and improve scientific rigor [59].

## 6. Future Prospects After ICH

### 6.1. Future Research Directions in Apoptosis and Neuroprotection

Research on apoptosis and neuroprotection holds promising potential for future developments in ICH. Mitochondrial dysfunction may offer new insights into hemorrhagic brain injury because mitochondrial stress can amplify apoptosis, oxidative damage, and energy failure after ICH [60]. Future research should focus on how mitochondrial pathways interact with ferroptosis, inflammatory injury, and blood‐brain barrier dysfunction and should identify therapeutic windows that are realistic for clinical intervention.

Future efforts should also clarify how inflammation intersects with neuroprotection and neuroregeneration after ICH [61]. For example, signaling pathways linked to Toll‐like receptor activation, cytokine release, and caspase activity may help explain why some anti‐inflammatory strategies reduce apoptosis, while others fail to translate clinically [62]. A more precise understanding of these interactions may support combination therapies rather than isolated pathway targeting.

### 6.2. Innovations and Breakthroughs in Neuroregenerative Technologies

Neuroregenerative technologies are poised for important innovations, but their relevance to ICH must be defined carefully. Three‐dimensional (3D) bioprinting may provide new tools for modeling the hemorrhagic brain microenvironment, testing biomaterials, and designing scaffold‐based delivery systems for stem cells or trophic factors [63]. In the future, these approaches may help improve localization, survival, and functional integration of therapeutic cells in damaged brain tissue.

Additionally, brain‐machine interfaces and other rehabilitation technologies may complement biological repair after ICH by improving motor and cognitive recovery [64]. However, such technologies should be regarded as adjuncts to stage‐specific neuroprotection and neurorestoration rather than substitutes for controlling the underlying secondary injury cascade.

## 7. Conclusion

In conclusion, apoptosis, neurogenesis, and stem cell therapy should be interpreted as interconnected components of the ICH neurorepair process rather than as separate topics [65]. Apoptosis remains central to secondary injury, but it interacts with ferroptosis, inflammatory signaling, and neurovascular dysfunction. Endogenous neurogenesis after ICH is biologically relevant yet limited, and current evidence for clinically meaningful human neuroregeneration remains insufficient. Stem cell therapy is promising because it may combine antiapoptotic, immunomodulatory, and proregenerative effects, but major translational barriers persist. Future progress will depend on preserving the neurovascular niche, distinguishing preclinical from human evidence, and designing rigorous, clinically relevant therapeutic strategies.

## Author Contributions

**Ziwei Wang**: conceptualization, writing – original draft. **Jing Tian**: investigation, writing – review and editing. **Ying Kong**: supervision, writing – review and editing, project administration.

## Funding

The authors declare that no funds, grants, or other support were received during the preparation of this manuscript.

## Disclosure

All authors contributed to the final manuscript revision and approval.

## Conflicts of Interest

The authors declare no conflicts of interest.

## Data Availability

Data sharing is not applicable to this article as no datasets were generated or analyzed during the current study.
